# Assessing the Impact of a Telemedicine Program on Caregivers of Children With Complex Chronic Conditions: Pre-Post Intervention Study

**DOI:** 10.2196/62953

**Published:** 2025-05-20

**Authors:** Aurora Madrid-Rodríguez, María José Peláez-Cantero, Alfonso Lendínez-Jurado, Ana Suárez-Carrasco, Elena Godoy-Molina, Antonia Gámez-Ruiz, Ana Corripio-Mancera, Yolanda Ramón-Telo, Ana García-Ruiz, Isabel Leiva-Gea

**Affiliations:** 1 Complex Chronic Patient and Palliative Care Unit, Department of Pediatrics, Regional University Hospital of Malaga, Málaga, 29590, Spain; 2 Instituto de Investigación Biomédica de Málaga y Plataforma en Nanomedicina-IBIMA Plataforma BIONAND, Calle Severo Ochoa, 35, Campanillas, Málaga, 29590, Spain, 34 952 36 76 00; 3 Universidad de Málaga, Andalucía Tech, Campus de Teatinos, Málaga, Spain; 4 Distrito Sanitario Málaga-Guadalhorce, Málaga, Spain

**Keywords:** telemedicine, telehealth, complex chronic children, pediatrics complex chronic conditions, palliative care, COVID-19, caregivers, children, pediatrics, chronic, SARS-Cov-2, pandemic, global health, remote consultation, quasi-experimental, quality of life, primary care

## Abstract

**Background:**

The COVID-19 pandemic was a paradigm shift in global health care. This situation highlighted the role of telemedicine in adapting to the care requirements of pediatric patients and their families by facilitating remote consultations and ensuring continuity of care.

**Objective:**

We aimed to establish the usefulness of a telemedicine program for caregivers of children with complex chronic conditions.

**Methods:**

We performed a quasi-experimental pre-post intervention study of a telemedicine program, regarding health care system use and caregiver quality of life while comparing two periods: before and during the COVID-19 pandemic. This study included caregivers of children with complex chronic conditions followed-up in a specialized unit.

**Results:**

A total of 34 families were included. The mean number of visits per year was higher in the preintervention period for both primary care consultations (*P*=.03) and hospital-specialized medical consultations (*P*=.03). The number of emergency room visits per year was lower in the pandemic period compared to the prepandemic period (*P*=.01). In both groups, an improvement in caregiver quality of life was detected at 12 months (*P*=.03). However, the Rosenberg self-esteem scale score for the primary caregiver was significantly lower at 3 months compared to the baseline (*P*=.03).

**Conclusions:**

Our study demonstrates that the use of a telemedicine program resulted in decreased scheduled face-to-face care visits and a reduction in the number of emergency department visits. Regarding the caregiver quality of life, outcomes were poorer among families whose children were affected by neurological conditions or were diagnosed at an older age.

## Introduction

Technological advances and progress in medicine have reduced infant mortality [1,2]. These survival successes have contributed to an increase in the prevalence of incurable diseases and disability, which leads us to reassess the way we approach and organize health services to adapt them to these new care needs [3,4].

In high-income countries, children with a complex chronic condition (CCC) represent 0.67%‐5% of the pediatric population; however, they consume up to 40% of total health expenditure, involving a high level of care both in and out of hospital, primary care, and non-health services [5-9].

The prevalence of life-threatening or life-limiting illnesses has increased in recent decades, with an estimated 61.1 per 10,000 children aged 1‐19 years in the United Kingdom. As reported, approximately 30% of these children have cancer; the remaining 70% comprise a combination of conditions, mainly neurodegenerative, metabolic, and genetic. Of these, 50% children will require the specialized care of a pediatric palliative care unit [10,11].

Therefore, a new strategy is required to transform the current care model, which is focused on treatment—where the patient is a passive subject—into a proactive model more centered on the needs of patients and their caregivers. In this revised model, the patients have more information and autonomy to play an active role in managing the disease [12,13].

In this context, new information and communication technologies (ICTs) provide tools that enable access to a wide range of resources. Numerous studies have endorsed telehealth as a supplementary approach to in-person care for managing chronic diseases [14]. However, the effectiveness of telehealth as a replacement for face-to-face care in patients with chronic conditions remains unclear [15,16].

In 2020, the COVID-19 pandemic transformed medical care through telemedicine with a demand for remote health care and telehealth [17,18]. While the world faced an unprecedented pandemic, patients with chronic diseases required special attention on an ongoing basis and, if warranted, an adaptation of their usual care [19]. This need arose as health care systems were overwhelmed, with most resources diverted to manage COVID-19 patients and their associated complications. The resulting strain on primary and hospital care made in-person evaluations for patients with chronic conditions especially challenging [20].

In complex chronic patient and pediatric palliative care units, telemedicine provides an accessible and equitable tool. With these tools, families can remain in contact with professionals by establishing a schedule for specific services in a time-effective manner and adapted to the stage of the illness [21,22].

Due to the COVID-19 pandemic and considering the vulnerability and fragility of these patients, some of the tools offered by ICT for the continuity of care have become even more useful, as they allow nonface-to-face visits; but with a capacity comparable to face-to-face visits, minimizing the risk of infection, and reducing the need to travel to a medical center [23]. Moreover, these tools may enhance treatment adherence, prevent medication errors, and foster greater engagement in self-care practices [24].

This study aimed to develop and evaluate a telemedicine program for the care of children with CCCs or those receiving palliative care, both before and during the COVID-19 pandemic, within a specialized pediatric palliative care unit.

## Methods

### Study Design and Setting

We designed a quasi-experimental pre-post intervention study in the Complex Chronic Patient and Palliative Care Unit of a pediatric tertiary hospital.

The study population comprised children with CCCs or those needing palliative care and their families. Inclusion criteria required that children meet the definition of medical complexity algorithm defined by the Seattle Children’s Hospital Group and score at least 6,5 points on the PedCom Scale [25,26]. Participants also had to be followed-up in the Complex Chronic Patient and Pediatric Palliative Care Unit of the hospital and had a high level of complexity, defined not only by the identification of the children as complex chronic patients but also by high consumption of resources: two or more hospital admissions, at least one admission to intensive care, or six or more visits to the emergency department in the last twelve months [27]. Patients older than 18 years or those whose families did not provide informed consent were excluded.

### Ethical Considerations

The study protocol was in accordance with the Declaration of Helsinki. All caregivers were informed about the study and signed a consent form, with no financial compensation provided. All data were anonymized to ensure confidentiality. This study was approved by the Research Ethics Committee of Malaga in May 2017 (reference: PIN-0287‐2016).

### Telemedicine Program Description

The telemedicine program was remotely managed and controlled by the hospital and its technical support center. It comprised three main functionalities:

1. Communication: The technology used was an Android-based smart TV platform connected to a television, which allows access to advanced telecommunication and medical device management services. It allows videoconferencing between patient (through the TV) and hospital (via PC), or between the patient and the medical professional (via PC, tablet, or smartphone), based on session initiation protocol technology (ie, market standard).
2. Information and training: Through the platform, families accessed a menu of audiovisual contents. The contents were developed by the professionals of the unit. This includes: information including telephone numbers of interest for caregivers; individualized access to videos, according to specific CCCs, with recommendations, procedures, theoretical and practical workshops including specific training such as nutritional management for patients receiving enteral support, oxygen therapy, or cardiopulmonary resuscitation, and PowerPoint presentations on neonatal techniques and care, secretion aspiration, and monitoring and respiratory care; and information on the current vaccination schedule.

1. Health care: The platform allowed clinical assessment via video call, with the option of requesting tests (laboratory and radiological), modification of technological support parameters, and issuing electronic medical prescriptions.

During recruitment, to avoid selection bias caused by the digital divide for families who did not have internet access at home, it was provided through project funding.

### Evaluation of the Program

The evaluation of the program was undertaken through the comparison of two one-year time periods; before the pandemic (March 2019 to February 2020) and during the pandemic (March 2020 to February 2021).

Sociodemographic variables described included age, gender, primary caregiver (parent or both parents), number of siblings, place of residence, and country of origin.

- Evaluation of face-to-face health care variables were categorized as follows: (1) scheduled: primary care consultations, hospital outpatient consultations per year, number of outpatient consultations in the Unit, number of home visits made by the Unit, number of scheduled hospital admissions; (2) urgent: number of emergency room visits, and number of urgent hospital admissions.

- Evaluation of telephone health care variables: (1) number of calls from the Unit to caregivers and (2) number of calls from caregivers to the Unit.

- Caregiver reported outcomes: (1) the Rosenberg Self-Esteem Scale [28,29], (2) the Positive and Negative Affect Schedule (PANAS) [30,31], (3) health-related quality of life of primary caregivers (EuroQol-5D-5L) [32], (4) short Zarit Caregiver Burden Interview (ZARIT) scale to assess primary caregiver burden [33,34].

The evaluation of the telemedicine program was carried out with periodic assessments (ie, baseline, 3, 6, and 12 months) of the health care variables and caregiver reported outcomes. The last assessment was carried out 12 months after the start of the intervention. The health care data was collected by telephone and the tests by mail and email.

### Data Analysis

Data analysis was performed using free R software (version 4.0.2; R Foundation for Statistical Computing). The Shapiro-Wilk test analysis was performed to determine the normality of the study variables. Data were presented as mean (SD) values in normal distributions or as median (IQR) for non-normal distributions. For independent quantitative variables, Student one-tailed *t* test was used for normally distributed data, and the Mann-Whitney *U* test for not normal distributed data. The Wilcoxon signed-rank test was performed to analyze differences in the non-normal distributions, and the paired *t* test for normal distributed data. To compare qualitative variables, the *χ*^2^ test was used for independent samples and McNemar test for related samples. A *P* value <.05 was considered statistically significant. *P* values were adjusted using the Benjamini-Hochberg correction to control for multiple comparisons.

## Results

### Study Population Characteristics

A total of 34 patients were included, ranging in age from 3 months to 14.2 years, with a median age of 4.9 years; 53% (18/34) were female participants. The most common CCC that affected our patients was neurological 73% (n=25), followed by gastrointestinal 71% (n=24), respiratory 50% (n=17), cardiovascular 41% (n=14), and oncological 21% (n=7). In addition, neurological CCCs were also the most frequent primary CCC in 47% (n=16), with cerebral palsy being the most prevalent neurological disease (11/16, 69%).

The primary caregiver was the mother in 82% (n=28) and 47% (n=16) of the families received social assistance; 91% of the families (n=31) received psychological care. A total of 29% (n=10) of the families were from a foreign country and 41% (n=14) had no other children; the number of children per family ranged from 0 to 5, with a median of 1.

### Impact of the Telemedicine Program on Health Care Activity During the Pandemic

Regarding scheduled care, bivariate analysis showed that the mean number of visits to primary care consultations per year was higher in the pre-pandemic period (*P*=.03), as well as more hospital specialty consultations (*P*=.03) (Table 1).

In terms of emergency care, there were significant differences in the number of emergency department visits per year during the compared to the prepandemic period (*P*=.01), with fewer visits during the pandemic. No significant differences were found in emergency hospital admissions between the two periods (Table 1).

Regarding telephone care provided by the Unit, we noted a nonsignificant increase in the number of calls made by the Unit during the pandemic (*P*=.06). However, there was a significant increase in telephone calls made by caregivers to the Unit during the pandemic compared to the pre-pandemic period (*P*=.003) (Table 2).

There were no significant differences between the mean length of hospital stays (measured in days) before and during the pandemic.

Tables1  2 display the variables related to the health care provided.

**Table 1.** Analysis of variables associated with face-to-face health care activity.

Type of care activity	Prepandemic period with telemedicine, mean (SD)	Pandemic period with telemedicine, mean (SD)	*P* value
Scheduled			
Primary care consultations	9.2 (9.9)	6.6 (5.9)	.03
Scheduled consultations hospital specialties	29.6 (19.2)	23.4 (19.2)	.03
Nurse consultations at CC^a^ and PPC^b^ Unit	8.3 (6.8)	10.1 (9.5)	.54
Pediatrician consultations at CC and PPC Unit	6.7 (5.6)	7.2 (5.6)	.77
Nursing home visits at CC and PPC Unit	3.1 (5.3)	2.4 (4.8)	.20
Pediatrician home visits CC and PPC Unit	2.8 (5.0)	2.1 (4.4)	.09
Scheduled admissions	0.6 (1.0)	0.6 (0.8)	.60
Urgent			
Emergency hospital visits	3.6 (4.0)	2.1 (2.1)	.01
Urgent hospital admissions	1.4 (1.5)	1.2 (1.5)	.19

a CC: complex chronic.

b PPC: pediatric palliative care

**Table 2.** Analysis of variables related to telephone health care activity.

Type of care activity	Pretelemedicine period, mean (SD)	Pandemic period with telemedicine, mean (SD)	*P* value
Unit calls to caregivers	36.4 (34)	48 (26)	.06
Caregivers calls to the Unit	6.2 (7.8)	10.5 (9.5)	.003

### Impact of the Telemedicine Program on the Caregiver-Reported Outcomes

Significant differences were seen in the visual analogue scale (VAS) of the EuroQol five-dimension (EQ-5D) quality of life test of the primary caregivers at 12 months compared with baseline, with an increase in VAS scores at 12 months (*P*=.03).

The Rosenberg self-esteem scale scores of the primary caregivers were significantly lower at 3 months than at baseline (*P*=.03). There was a significant negative correlation between the short Zarit Caregiver Burden Interview (ZARIT) scale for primary caregiver burden at 6 months and the number of siblings (adjusted *R*^2^=0.37; *P*=.009). The VAS score of the EQ-5D quality of life scale for primary caregivers at 12 months correlated negatively and significantly with age at diagnosis of the child’s illness (adjusted *R*^2^=0.24; *P*=.04).

The difference in the index value of the EQ-5D quality of life scale for primary caregivers from baseline to 3 months was negatively correlated with the age at patient admission (adjusted *R*^2^=0.97; *P*=.007).

In the analysis of the relationship between caregiver quality of life and the different types of CCCs, the difference in index value of the EQ-5D quality of life scale for primary caregivers from baseline to 3 months was negatively correlated with neurological disease (adjusted *R*^2^=0.89; *P*=.03).

Table 3 shows the results of the tests at baseline and at 3, 6, and 12 months.

**Table 3.** Analysis of quality of life test results.

Quality of life tests	0 months, mean (SD)	3 months, mean (SD)	6 months, mean (SD)	12 months, mean (SD)	*P* value
Rosenberg self-esteem scale	29.3 (5.2)	27.3 (6.4)	31.0 (4.2)	33.0 (4.8)	.03
Positive and negative affectivity scales (PANAS)					
Positive affect	29.1 (7.4)	29.5 (7.9)	33.1 (9.4)	33.8 (7.5)	.41
Negative affect	26.8 (8.8)	23.8 (6.8)	24.3 (7.1)	22.8 (6.2)	.34
Health-related quality of life (EuroQol-5D-5L)					
EQ-5D^b^ value	0.814 (0.180)	0.766 (0.151)	0.838 (0.153)	0.838 (0.142)	.053
EQ-5D VAS score	76 (18)	75 (15)	78 (14)	75 (21)	.03
Short ZARIT^a^ scale	20.1 (7.0)	24.3 (5.0)	20.8 (5.9)	19.8 (5.7)	.44

a ZARIT: Zarit Caregiver Burden Interview.

b EQ-5D: EuroQol-five dimension.

## Discussion

### Principal Findings

The development of telemedicine programs has transformed care for patients with chronic illnesses or those in palliative situations. Several studies including the one by Prabhakaran et al [35], highlights the effectiveness of a mobile health intervention in primary care settings within rural Indian communities. This initiative focused on preventing and managing cardiometabolic conditions, depression, and related risk factors among adults. In pediatrics, evidence-based studies, including the consensus developed by the Italian Societies of Telemedicine, Preventive and Social Pediatrics, and Pediatric Primary Care, among others, have demonstrated the critical role of telemedicine in managing patients with chronic conditions (eg, cardiology, respiratory, or neurology) or those requiring palliative care. These findings underscore the potential of telemedicine to enhance healthcare delivery by facilitating improved collaboration among multidisciplinary professionals and patients, creating innovative opportunities to optimize clinical outcomes and service quality [36].

In our study, the most common condition was neurological, which is the most frequent condition as described in literature indicating that neurological patients are the most prevalent. Our findings are in accordance with prior research, with neurological CCCs being the most frequent, followed by gastrointestinal and respiratory CCCs. Also coinciding with published findings, the mother most often fulfilled the role of the primary caregiver [13,37-41].

During the COVID-19 pandemic, as seen in other studies, a decrease was observed in both scheduled and urgent care for children with CCCs, similar to trends in the general pediatric population and among children with special health care needs [42-49].

The increase in telephone consultations during the pandemic, both initiated by the Unit and caregivers, especially the increase in calls from caregivers, may be explained by the decrease in face-to-face care in primary care and in specialized hospital care during this period. This finding highlights the support provided by the Unit via telephone to minimize the impact of the pandemic on children with CCCs, as described in several studies focused on specific care strategies aimed at this particularly vulnerable population [50].

In our study, the absence of significant differences in scheduled or urgent hospital admissions, has been described in other studies on telemedicine in children with CCCs [22]. This may be due to the high complexity of the patients included in this study, where pandemic-related factors may not have influenced the number of hospital admissions or the length of hospital stays.

Several studies have described the importance of the psychosocial needs of children with CCCs and their families and how these needs, especially concerning mental health increased during the pandemic [51,52]. To date, few studies have demonstrated improvements in the quality of life of caregivers from telemedicine programs, often due to the cross-sectional design of studies without multiple cut-off points. Our study highlights the importance of conducting studies that monitor the impact and benefits of these programs throughout the intervention to verify their usefulness [53-55].

In our study, a greater burden on primary caregivers was detected in families with more children, a demographic factor not previously described and one that should be considered to reinforce support for these families.

The quality of life of the primary caregivers measured by the VAS value of the EQ-5D scale at 12 months was poorer in those with children with CCCs diagnosed at an older age. Similarly, we found a negative correlation between the difference in the EQ-5D quality of life scale of the primary caregivers at 3 months, compared to baseline in children with CCCs who were older at admission to the Unit. Our analysis revealed that both the child's age at diagnosis and age at Unit admission were inversely associated with parental quality of life, showing the most pronounced negative impact at 3- and 12-month follow-up during the pandemic. The influence of the age of the child with a CCC on the quality of life of the primary caregivers during the pandemic has not been examined previously and merits further investigation as a potential modulator of the quality of life of the parents.

Published studies on the impact of neurological diseases have shown an increased family, work, and economic burden on primary caregivers, specifically on caregivers of children with cerebral palsy [56]. In our study, a negative correlation was found between the index value of the EQ-5D quality of life scale of the primary caregiver at 3 months, compared to baseline with neurological CCCs, which was also the most frequent CCC observed.

The observed lower self-esteem among the primary caregivers at 3 months of the telemedicine program during the pandemic setting may be explained by the effects of full confinement, including reduced psychosocial and family support, adaptation of home care with a change in care support, and the increased care burden. This reduction in the quality of life of the primary caregiver at 3 months has not been evaluated in other studies and may be useful for reinforcing psychosocial support to families, considering this period as a turning point in caregiving. Conversely, the observed improvement in VAS of the EQ-5D quality of life test may reflect an increased quality of life at 12 months, which could be attributed to adaptation to the situation as well as decreased isolation measures one year after the pandemic.

It is important to leverage the growth of the ICTs during the pandemic for the benefit of patients, especially those who are most vulnerable and with specific care needs such as children with CCCs. Telemedicine provides equity in care and efforts should be made to evaluate the impact of telemedicine programs in patients, primary caregivers, and health care teams, taking into account the clinical, economic, and quality of life variables in patients and their families monitored over time.

### Limitations

The use of telemedicine prior to the pandemic was demonstrated to be effective in children with CCCs, both in reducing the number of emergency department visits and costs [55,57-61]. However, in our study, it was challenging to determine with certainty whether the decrease in urgent or unscheduled care could be attributed to the COVID-19 pandemic or the telemedicine program intervention. During the pandemic, there was a decrease in emergency department visits, as many caregivers were afraid to go to hospitals to avoid infections in their children [43,45]. In addition, we must take into account the decrease in respiratory symptoms due to the confinement and isolation measures described in several studies [45,47,49]. Studies with larger and more homogeneous samples should be carried out after the pandemic to assess whether its effectiveness in terms of care is maintained without the interference caused by the pandemic.

### Conclusions

In our study, the use of a telemedicine program during the pandemic resulted in a decrease in scheduled face-to-face care and a reduction in the number of emergency department visits compared to the prepandemic period. Conversely, we observed an increase in telephone support without any impact on hospital admissions. We identified the most vulnerable families in this group of patients, including those with a child affected by a neurological condition, those who had an older child when the disease was diagnosed, and families with a greater number of children. These insights allowed could help guide the redirection of limited resources. Our findings indicated that the telemedicine program was effective in the supporting care of children with CCCs and their families.

## Abbreviations

CCC: complex chronic condition
EQ-5D: EuroQol five-dimension
ICT: information and communication technologies
VAS: visual analogue scale
